# SARS-CoV-2 evolution in animals suggests mechanisms for rapid variant selection

**DOI:** 10.1073/pnas.2105253118

**Published:** 2021-10-29

**Authors:** Laura Bashor, Roderick B. Gagne, Angela M. Bosco-Lauth, Richard A. Bowen, Mark Stenglein, Sue VandeWoude

**Affiliations:** ^a^Department of Microbiology, Immunology, and Pathology, Colorado State University, Fort Collins, CO 80523;; ^b^Department of Pathobiology, Wildlife Futures Program, University of Pennsylvania School of Veterinary Medicine, Kennett Square, PA 19348;; ^c^Department of Biomedical Sciences, Colorado State University, Fort Collins, CO 80523

**Keywords:** SARS-CoV-2, viral variants, companion animals, host adaptation, spillover

## Abstract

SARS-CoV-2 spillback from humans into domestic and wild animals has been well documented, and an accumulating number of studies illustrate that human-to-animal transmission is widespread in cats, mink, deer, and other species. Experimental inoculations of cats, mink, and ferrets have perpetuated transmission cycles. We sequenced full genomes of Vero cell–expanded SARS-CoV-2 inoculum and viruses recovered from cats (*n* = 6), dogs (*n* = 3), hamsters (*n* = 3), and a ferret (*n* = 1) following experimental exposure. Five nonsynonymous changes relative to the USA-WA1/2020 prototype strain were near fixation in the stock used for inoculation but had reverted to wild-type sequences at these sites in dogs, cats, and hamsters within 1- to 3-d postexposure. A total of 14 emergent variants (six in nonstructural genes, six in spike, and one each in orf8 and nucleocapsid) were detected in viruses recovered from animals. This included substitutions in spike residues H69, N501, and D614, which also vary in human lineages of concern. Even though a live virus was not cultured from dogs, substitutions in replicase genes were detected in amplified sequences. The rapid selection of SARS-CoV-2 variants in vitro and in vivo reveals residues with functional significance during host switching. These observations also illustrate the potential for spillback from animal hosts to accelerate the evolution of new viral lineages, findings of particular concern for dogs and cats living in households with COVID-19 patients. More generally, this glimpse into viral host switching reveals the unrealized rapidity and plasticity of viral evolution in experimental animal model systems.

## Significance

SARS-CoV-2 emerged because of viral spillover from animals to humans, and spillback to other animal species has been observed with accelerating frequency. Cross-species transmission generally results in the rapid adaptation of the virus to the new host, and repeated transmissions may hasten viral evolution and novel strain emergence. We report the surprisingly rapid selection of numerous SARS-CoV-2 variants in cell culture and following infection of nonhuman mammalian hosts, including dogs and cats. These molecular changes in SARS-CoV-2 provide insight into mechanisms of viral host adaptation, lay the groundwork for additional studies assessing dominant variant fitness and phenotype, and highlight the potential for human reinfection with new viral variants arising in species in close and frequent contact with humans.

Cross-species transmission events, which challenge pathogens to survive in new host environments, typically result in species-specific adaptations (1). These evolutionary changes can determine the pathogenicity and transmissibility of the virus in novel host species (2). Pathogen host switching resulting in epidemic disease is a rare event that is constrained by the interaction between species (3). In contrast to most species, humans move globally and regularly come into contact with domestic and peridomestic animals. Thus, when a novel virus spreads through human populations, there is an incidental risk of exposure to potentially susceptible nonhuman species.

This scenario has become evident with the SARS-CoV-2 pandemic (*SI Appendix*, Table S1). Originally resulting from viral spillover into humans (4, 5), likely from an animal reservoir, spillback into a wide range of companion and wild animals has occurred or been shown to be plausible (6–10), and an increasing number of studies have indicated a high frequency of human-to-animal SARS-CoV-2 spillback transmission (11–18). Given the short duration of viral shedding, serologic analyses present a more accurate characterization of actual animal exposures to SARS-CoV-2. Such studies conducted in a variety of animal species have illustrated surprisingly high levels of seroconversion in cats and dogs and more recently free-ranging deer (*SI Appendix*, Table S1) (73–87). Other well-documented spillback events include numerous mink farms (*SI Appendix*, Table S1). In one of these reports, multiple feral cats living on a mink farm in the Netherlands during a SARS-CoV-2 outbreak were seropositive, likely from the direct transmission of the virus from mink to cats, as owned cats on the same farm were seronegative (19). This further illustrates that cross-species transmission chains are readily achieved. Recent surveys of free-ranging white-tailed deer in Illinois, Michigan, New York, and Pennsylvania revealed 33% seropositivity in free-ranging animals (20). Active SARS-CoV-2 infection was subsequently confirmed by PCR in a deer in Ohio (21). Together, these findings suggest the likely establishment of multiple domestic animal and wildlife reservoirs of SARS-CoV-2.

The repeated interspecies transmission of a virus presents the potential for the acceleration of viral evolution and a possible source of novel strain emergence. This was demonstrated by reverse zoonosis of SARS-CoV-2 from humans to mink, followed by a selection in mink and zoonotic transmission back to humans (8). Given that reverse zoonosis has been reported repeatedly in dogs and cats from households where COVID-19 patients reside, and the fact that up to 50% of households worldwide are inhabited by these companion animals, there is potential for similar transmission chains to arise via humans and their pets (22, 23). Elucidating the viral selection and species-specific adaptation of SARS-CoV-2 in common companion animals is therefore of high interest. Furthermore, understanding viral evolutionary patterns in both companion animals and experimental animal models provides a valuable appraisal of species-specific viral variants that spotlight genomic regions for host–virus interaction.

Significant attention has been directed at substrains evolving from the initial SARS-CoV-2 isolate (24), and an accumulating number of variant lineages have demonstrated increased transmission potential in humans (25, 26). The role, if any, that reverse zoonotic infections of nonhuman species and spillback may have played in the emergence of these novel variants of SARS-CoV-2 remains unknown. Documenting viral evolution following the spillover of SARS-COV-2 into new species is difficult given the unpredictability of timing of these events; therefore, experimental studies can greatly aid the understanding of SARS-CoV-2 evolution in animal species. Laboratory-based studies also provide the opportunity to determine how changes that occur during viral expansion in cell culture may influence in vivo infections. This information is highly relevant for the interpretation of in vivo and in vitro experiments using inoculum propagated in culture.

We therefore assessed the evolution of SARS-CoV-2 during the three rounds of expansion of strain USA-WA1/2020 in Vero E6 cells (27), followed by measuring the variant emergence occurring during primary experimental infection in four mammalian hosts. Specifically, we compared variant proportions, insertions, and deletions occurring in genomes of SARS-CoV-2 recovered from dogs (*n* = 3), cats (*n* = 6), hamsters (*n* = 3), and a ferret (*n* = 1).

## Results

### SARS-CoV-2 Genome Sequence Recovery.

We recovered full-genome sequences of SARS-CoV-2 from stocks of three serial passages of USA-WA1/2020 inoculum and viruses recovered from five cats with primary exposure, one cat exposed by contact to an infected cat (6), three dogs (6), three hamsters, and one ferret (*SI Appendix*, Table S2). PCR amplification of the full–SARS-CoV-2 genome failed in two additional ferrets because of low viral titers; these samples were subjected to the targeted PCR of spike sequences followed by Sanger sequencing. We obtained a median depth of coverage of 2,859× (mean = 4,380×) using ARTIC version 3 primers, as described in *Materials and Methods* (*SI Appendix*, Fig. S1). SARS-CoV-2 genomes recovered from Cat 5 and one technical replicate of Cat 4 were amplified with ARTIC version 2 primers with median depth of coverages of 506× and 1,056×, respectively.

### Identification of Single-Nucleotide and Structural Variants.

**Fig. 1.**
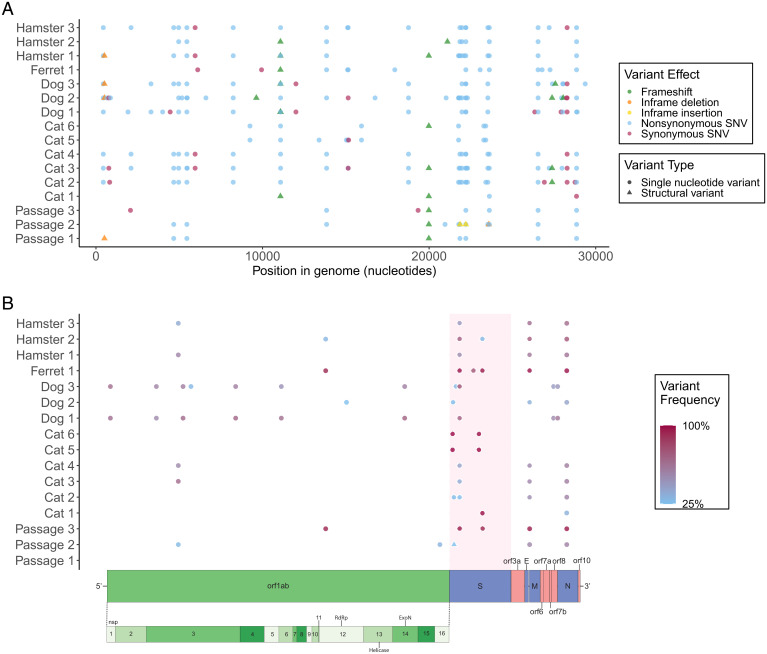
A total of 88 unique variants were detected in ≥3% of sequences of in vitro– and in vivo–derived SARS-CoV-2. The position, predicted effect (*A*), and allele frequency (*B*) of SNV and SV variants detected across the SARS-CoV-2 genome in sequences obtained from 13 experimentally inoculated animals and three passages of the viral inoculum. Each point represents an SNV (circle) or SV (triangle). (*A*) All variants detected in ≥3% of sequences, demonstrating a majority of SNVs and a slightly increased occurrence of modifications in the spike protein. (*B*) All variants detected in ≥ 25% of sequences, revealing the presence of higher-frequency variants in the spike protein of all datasets, excluding the P1 stock virus, and the prevalence of higher-frequency variants across the entire genome of SARS-CoV-2 recovered from dogs. The frequency is indicated by scale from blue (low) to red (high). The schematic of the SARS-CoV-2 genome is illustrated for orientation.

A total of 88 unique single-nucleotide (SNV) and structural variants (SV; insertions and deletions) were present in 3 to 100% of viral genome sequences in two technical replicates and were observed 270 times across all datasets (242 SNVs and 28 SVs; Fig. 1*A*). This included 74 SNVs and 14 SVs. A total of 71 of 270 variants (26.3%) were detected at 25% frequency or greater (Fig. 1*B*). A total of 70 of the 88 unique variants (79.5%) were not detected in any of the three viral inoculum stocks at greater than or equal to 3% frequency. Seven SARS-CoV-2 SNVs, recovered from one or more species, occurred at positions that coincide with variants of concern in humans or other species (Table 1).

**Table 1.** Seven SARS-CoV-2 SNV recovered from one or more species have been reported as variants of concern in humans or other species

Position	CDS	Variant	Species	P1/P2/P3 inoculum frequency (%)	Reported variant(s)	Reported species
21768	Spike, NTD	H69R	Ferret, cat, dog, hamster	2.1/1.2/0	69–70del	Human (25), mink (40)
22205	Spike, NTD	D215H	Cat, dog, ferret, hamster	4.2/19.4/96.6	D215G	Humans (42), deer mice (43)
22205		D215N	Cat, dog, hamster	1.4/1.5/0		
23064	Spike, RBD	N501T	Ferret	0/0/0	N501T	Ferret (39), mink (40)
					N501Y	Human (25, 42), BALB mice
23403	Spike	D614G	Cat	0/0/0	D614G	Human (54)
23525	Spike	H655Y	Cat, dog, ferret, hamster	1.5/4.8/1.1	H655Y	Cat (52), human (54), hamster (72)
23618	Spike	S686G	Cat	0/0*/0	S686G	Ferret (39)
11083	orf1ab, nsp6	L37F	Cat, dog, hamster	1.8/1.8/0*	L37F	Human (54)

The frequency of each variant detected in Vero E6 cell supernatant at each passage (P1, P2, and P3) represents the average of two replicates; values undetected above 0.1% are reported as 0. Only D215H was detected at high frequency in P3 inoculum.

*S686G was detected in one replicate of P2 at 0.8% and undetected in the second; L37F was detected in one replicate of P3 at 0.5% and undetected in the second.

### Cell Culture–Associated Viral Variants and Reversion in Animals.

**Fig. 2.**
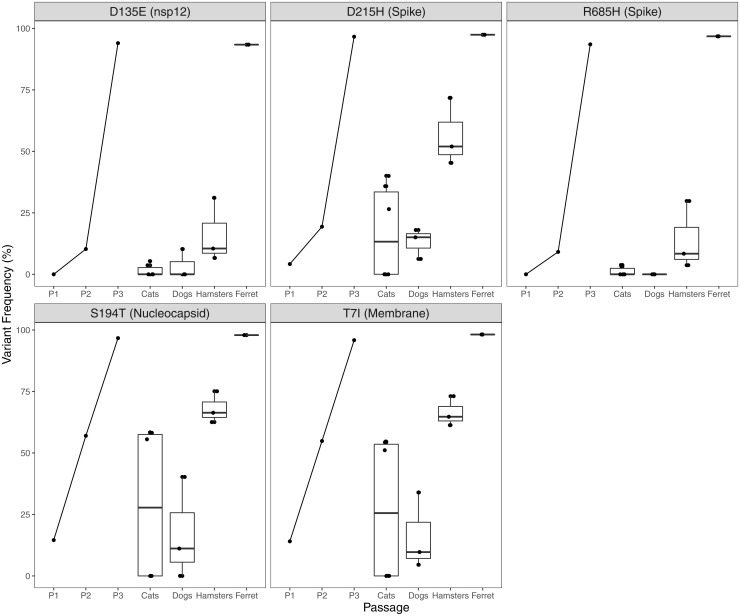
SARS-CoV-2 cell culture variants revert rapidly during in vivo experimental infection. SARS-CoV-2 isolate USA-WA1/2020 was passaged three times in Vero E6 cell line. Five SNV substitutions across the genome reached >93% frequency; the variant proportion recovered from each supernatant stock is indicated by P1, P2, and P3. Cats, dogs, hamsters, and the ferret (*n* = 6, 3, 3, and 1, respectively) were inoculated with 10^5^ to 10^6^ pfu intranasally. Virus was recovered 1 to 3 d postinoculation, sequenced using a tiled amplicon technique and analyzed with a pipeline for calling SNVs and SVs in viral populations. Cell culture variants decreased in frequency in all animals with the exception of the ferret (*n* = 1). Variants are indicated in the reference to the consensus residue in USA-WA1/2020 and their position within the coding sequence of a SARS-CoV-2 protein. Each point represents the mean of two technical replicates, aside from one cat, for which a replicate was not sequenced.

The passage of SARS-CoV-2 in Vero E6 cells resulted in five nonsynonymous amino acid changes that reached fixation or near fixation following three passages (Fig. 2). These variants reverted to wild-type sequences within 1- to 3-d after inoculation in dogs, cats, and hamsters. Reversion frequency was higher in samples recovered 3-d postinfection (dogs and cats) compared to a virus recovered 1-d postinfection (hamsters; Fig. 2). Spike variant D215H underwent a second substitution to D215N, which reached near fixation (>72%) in two dogs. This variant was also detected at >6% frequency in the third dog, one hamster, and three cats.

### Viral Variants Arising during In Vivo Passage.

A total of 13 nonsynonymous and one synonymous variant (*n* = 14 emergent variants) not present in the USA-WA1/2020–derived viral inoculum at ≥3% frequency, were detected at >50% frequency in one individual or were detected in all individuals of one species (Table 2). The default limit of detection for variant calling was set at 3%, but it was possible to observe variants with frequencies down to 0.1%, with possibly reduced quantitative accuracy (28). There were 564 unique variants detected at 0.1% frequency or greater across all 16 datasets in the study. Of these, 174 were detected in the three cell culture–derived samples, and 390 (69%) were not identified in the viral inoculum at all. Variants detected at lower frequencies within the Passage 3 (P3) inoculum in matched replicates would be suggestive of the in vivo selection of these preexisting quasispecies. Using this reasoning, we detected 10 of the 14 emergent variants from animal hosts at 0.1 to 3% in the viral inoculum, and four were not identified in the viral inoculum at all.

**Table 2.** Nonsynonymous SARS-CoV-2 variants emerge following experimental inoculation in cats, dogs, hamsters, and the ferret

Variant	Position	CDS	N or S	Cat 1	Cat 2	Cat 3	Cat 4	Cat 5	Cat 6	Dog 1	Dog 2	Dog 3	Ferret 1	Hamster 1	Hamster 2	Hamster 3
G59D	441	nsp1	N		0.15	0.06	0.10			**0.66**	0.14	**0.66**		0.04		0.07
E195G	3303	nsp3	N							**0.53**		**0.56**				
T749I	4965	nsp3	N		0.16	0.06	0.10			**0.68**	0.13	**0.59**			0.04	0.07
H1841Y	8240	nsp3	N		0.17	0.06	0.10			**0.70**	0.13	**0.59**		0.04		0.07
L37F	11083	nsp6	N		0.15	0.06	0.09	0.05	0.06	**0.64**	0.10	**0.49**		0.04		0.06
I242V	18763	nsp14	N		0.18	0.06	0.10			**0.71**	0.15	**0.58**				0.06
H69R	21768	S	N		0.06			**1.00**	**0.99**		0.14			0.06		
D138Y	21974	S	N		0.07	0.06	0.05			0.06	0.07	0.29		0.10	0.12	0.10
D215N	22205	S	N		0.17	0.07	0.10			**0.72**	0.14	**0.78**				0.06
N501T	23064	S	N										**0.76**			
D614G	23403	S	N		0.16			**0.99**	**0.98**							
S686G	23618	S	N	**0.99**												
S43Y	28021	orf8	N							**0.53**		0.44				
N4N	28285	N	S		0.15	0.06	0.11			**0.62**	0.10	**0.52**				0.05

SARS-CoV-2 isolate USA-WA1/2020 was expanded for three passages in Vero E6 cells and 10^5^ to 10^6^ pfu was inoculated intranasally in cats, dogs, hamsters, and the ferret (*n* = 6, 3, 3, and 1). Virus was recovered 1- to 3-d postinoculation (dog and cat infections described in ref. 6), sequenced using a tiled amplicon technique and analyzed with a pipeline for calling SNVs and SVs in viral populations. Variants representing >50% of recovered genomes in at least one individual or that were found in all individuals of a species are indicated here. Bold indicates variants occurring in >50% of genomes; boxes indicate variants detected in all sampled individuals of that species. Only one synonymous mutation met the criteria and is displayed. Variants that emerged following the passage in Vero E6 cells (Fig. 2) are not listed. Each point represents the mean of two technical replicates, aside from Cat 5, for which a replicate was not sequenced.

**Fig. 3.**
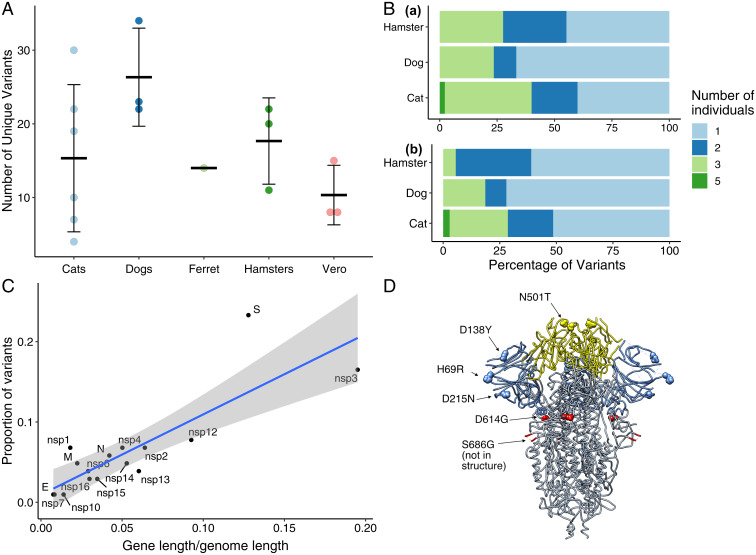
SARS-CoV-2 viral evolution differs across species, gene regions, and individuals. (*A*) Each point indicates the number of unique variants detected at ≥3% frequency in SARS-CoV-2 genomes recovered from individual animals. There is no significant difference in the number of unique variants detected in different species (ANOVA, *P* = 0.22). (*B*) Analysis of variant distribution within species reveals that the majority of variants were detected in just one individual within each species. Subplot *a* shows the distribution for all variants, while *b* illustrates only variants not occurring in the P3 inoculum at ≥3%. (*C*) Variants are distributed across viral genes in relation to each gene’s length as a proportion of the entire genome length (linear model, *R*^2^ = 0.69, and *P* < 0.0001). The spike protein contained a notably higher proportion of all variants in comparison to its share of genome length. Gray shading represents the 95% CI for the slope of the regression line. (*D*) SARS-CoV-2 spike protein variant “residues of concern” are in NTD, RBD, and furin cleavage site. Residues described in the main text and Table 1 in the SARS-CoV-2 spike trimer are highlighted on structure 6VXX. Blue indicates NTD and yellow indicates RBD. The furin cleavage site and adjacent residue 686 are in the indicated loop, which was not resolved in this structure.

There was no significant difference between the mean number of unique variants detected in viral genomes recovered from the four species (Fig. 3*A*). Viral titer did not seem to determine the observed variant richness. We were not able to detect a viable virus at 3-d postinfection in any of the dogs (<1 log plaque-forming units [pfu]/mL); however, we observed 24, 34, and 22 variants in Dogs 1, 2, and 3, respectively, which is in the same range as Cats 2, 3, and 4, for which we assessed viral titers of 5.3, 6, and 5.4 log pfu/mL and 22, 30, and 19 variants, respectively. In addition, Hamster 1 had 20 variants and a titer of 3.3 log pfu/mL, whereas Hamster 3 had 22 variants and a titer of <1 log pfu/mL.

SARS-CoV-2 sequences also did not cluster by species (*SI Appendix*, Fig. S2). Excluding variants that were detected in the original inoculum sequences at ≥3% frequency, 69.6% of novel variants were found in a single species, 11.6% were found in two species, and 17.4% were found in three species. Only one variant that was not detectable in the inoculum at ≥3% frequency was detected in all four animal species, a single-nucleotide insertion at L37 in nsp6 causing a frameshift.

Variants were unevenly distributed among individuals of a species. When variants detected in the viral inoculum were excluded from the analysis, a large proportion of variants detected in cats, dogs, and hamsters were observed in only one individual of the species (Fig. 3*B*). The SARS-CoV-2 spike gene contained a larger proportion of variants relative to its gene length (Fig. 3*C*), and the majority of variant positions corresponding to human variants of concern were in the spike protein (Table 1 and Fig. 3*D*).

Six nonsynonymous and one synonymous amino acid variant were found in all dogs and were present in >50% of sequences recovered from two of three dogs (Table 2). These seven variants were found in cats and hamsters at lower frequency. A single novel amino acid variant (D138Y) was detected in all three hamsters. Cats 1 to 5 were experimentally inoculated; Cat 6 was infected through cohousing with Cat 5 1-d postinfection (6). Cats were inoculated in two cohorts (Cohort 1 = 1, 2, and 3 and Cohort 2 = 4, 5, and 6). The virus recovered from cats in Cohort 1 had significantly more variants than cats in Cohort 2, but variants were at higher frequency (near fixation) in cats in Cohort 2 (*P* < 0.02). This observed variation is likely due to the difference in the sample collection timeline between the two experimental cohorts. Two amino acid variants (H69R and D614G) were found at >98% in Cats 5 and 6, and S686G was found in 99% of sequences from Cat 1. Around 6 of the 10 variants detected in Cat 5, including spike variants H69R and D614G, were also detected in Cat 6, which was infected through contact with Cat 5 (*SI Appendix*, Table S3). The single unique variant detected in the contact cat caused a frameshift at D124 in nsp15. The depth of coverage for Cat 5 was <40× at this position, so no variants were called. No amino acid variants from SARS-CoV-2 sequences were shared across all six cats.

We detected spike variant N501T in Ferret 1 at a frequency of 76%. Two additional ferret nasal wash samples from the same experimental cohort were subjected to targeted PCR, cloning, and direct sequencing to ascertain the presence of this variant in additional ferrets. N501T (A > C at position 23064 in USA-WA1/2020) was present in four of the six (66.7%) clones from Ferret 2 and two of the three (66.7%) clones from Ferret 3.

### Signatures of Selection.

**Fig. 4.**
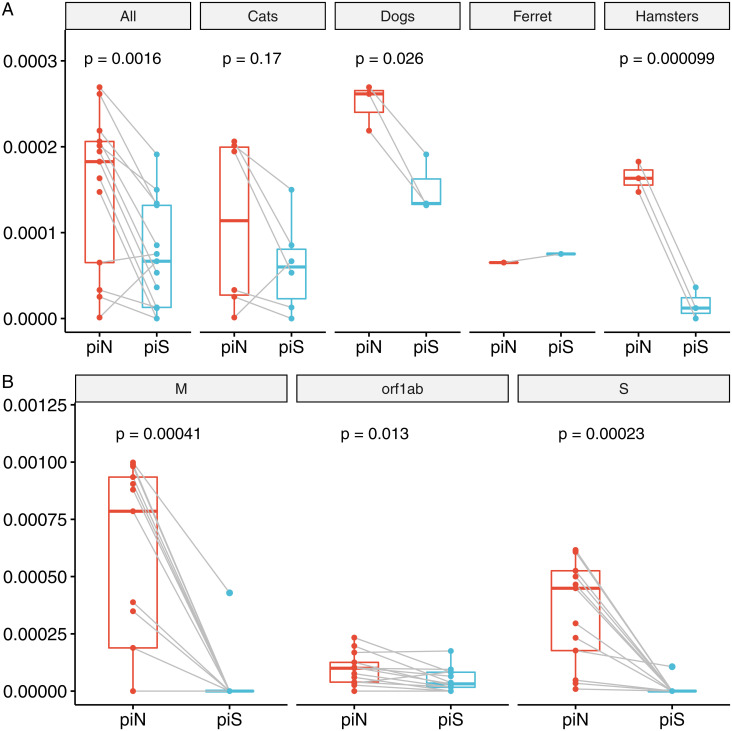
Signatures of positive selection are detected in SARS-CoV-2 genome sequences recovered from experimentally inoculated cats, dogs, hamsters, and the ferret. (*A*) Comparison of nonsynonymous (piN) and synonymous (piS) nucleotide diversity reveals that piN is significantly greater than piS, indicating positive selection. Each point represents a measurement of piN (red) or piS (blue) calculated for the entire SARS-CoV-2 genome from sequences recovered from an individual animal, relative to the reference sequence of USA-WA1/2020. The analysis of the same measures within each species reveals that piN > piS for viral genomes is greater in dogs and hamsters. (*B*) Orf1ab, S, and M are undergoing positive selection in mammalian hosts. Each point represents piN or piS calculated for a specific SARS-CoV-2 gene or open reading frame from sequences recovered from an individual animal.

Nonsynonymous (πN) and synonymous (πS) nucleotide diversity and πN/πS for SARS-CoV-2 populations were evaluated as a measure of within-host diversity and signatures of selection (Fig. 4 and *SI Appendix*, Tables S4 and S5). At the population level, πN was greater than πS for 11 out of 13 SARS-CoV-2 samples (paired *t* test, *P* < 0.01), indicating positive selection across hosts (Fig. 4*A*). At the species level, the selection of SARS-CoV-2 in dogs and hamsters was most significant (Fig. 4*A*). Furthermore, we recorded a significant difference between πN and πS in orf1ab, spike, and membrane proteins (*P* < 0.02, Fig. 4*B*), indicating a positive selection at the level of these gene products. In particular, spike πN was significantly greater than πS for viral genomes recovered from all 16 host and cell culture samples (*P* < 0.0001).

## Discussion

### Cell Culture–Associated Mutation.

Variants that reached near fixation in cell culture but reverted to wild type in animal infections suggest a role for these residues in facilitating in vivo versus in vitro infection. The rapid reversion of variants arising in Vero E6 cells (originally derived from African green monkeys) occurred in dogs, cats, and hamsters, suggesting that selective advantages of cell culture variants are due to differences between in vivo and in vitro environments rather than specific host–virus adaptation. Alternatively, changes may reflect host-specific adaptations to the monkey and not in vitro infections. This explanation is less likely as the variants reverted in multiple species that are more distantly related to humans than African green monkeys, and distinct differences exist between in vivo and in vitro environments. For example, in vivo viral attachment and entry require the circumvention of respiratory epithelial defenses, such as mucus and cilia; residues which play roles in overcoming these physical features would not be highly selected in cell culture.

Amino acid R685 is located in the furin cleavage site, a motif unique to SARS-CoV-2 relative to SARS-CoV and other SARS-related coronaviruses (29). The PRRAR insertion at this location provides a motif for serine proteases to cleave the spike protein into S1 and S2 subunits, a key step to initiating viral entry via membrane fusion. The deletion of the furin cleavage site has been shown to reduce SARS-CoV-2 replication in human respiratory cells, hamsters, and hACE2 mice (30). Previous work has demonstrated the rapid emergence of spike variants at the furin cleavage motif, including R685H, following five passages in type II transmembrane serine protease (TMPRSS2)–deficient cells (31). This explains the emergence of this variant in our study following the propagation in Vero E6 cells, which do not express high levels of TMPRSS2 (32). The distribution of variants arising in vitro (nsp12, spike, N, and M; Fig. 2) indicates functions beyond receptor binding and entry as drivers of selection, which would be predicted to occur in spike.

### Patterns in Viral Evolution within and across Species.

We observed the emergence of low-, intermediate, and high-frequency variants across the SARS-CoV-2 genome in viral RNA recovered from cats, dogs, hamsters, and ferrets. The abundance of variants detected in our study is in contrast to the few variants observed in large datasets of SARS-CoV-2 genomes recovered from humans (33). Low-frequency variants (<25%) may represent mutations occurring during population expansion, following the infection of a new individual. The majority of variants arising in vivo were in the spike protein, which was under the strongest selective pressure, illustrating the importance of this residue in shaping cross-species transmission events. Variants detected in animals were likely to arise either by the rapid selection of low-frequency inoculum variants with structural features that favored in vivo attachment, entry, and replication or as de novo mutations arising from errors during viral replication.

The magnitude of cell culture variant reversion was greater in dogs than other species. All five cell culture–associated variants decreased from >93 to <41%; in particular, D135E and R685H decreased to ≤10%. SARS-CoV-2 was not culturable from canine samples, consistent with previous reports (34), and was below the limit of detection by qPCR, though low-level seroconversion suggested some viral replication had occurred (6). These experimental results contrast with widespread global reports of canine companions becoming infected through contact with their SARS-CoV-2–infected owners (35–37). Many variants detected in dogs were not found in the inoculum, reached high frequencies (Table 2), and were under strong selective pressure (Fig. 4). The majority of canine variants were found in nonstructural replicase genes (Table 2), providing strong evidence of selective pressure and host–viral molecular interactions in dogs directed toward the selection for viral replication function. The differences between reports of natural reverse zoonotic infections and laboratory exposures in dogs may stem from disparate doses, dose frequency, or strains (human-adapted or cell culture–propagated virus).

Both domestic and nondomestic cats appear to be highly susceptible to SARS-CoV-2 infection and readily transmit the virus to other cats (6–8). The similarity between sequences recovered from Cat 5 and Cat 6 (exposed via direct contact with Cat 5) demonstrates the ability of novel mutations to be horizontally transmitted (*SI Appendix*, Table S3). In contrast to dogs, high viral titers were recovered from cats (6), and fewer consensus and shared variants arose in cats than dogs, suggesting that SARS-CoV-2 viral pathogenesis in cats is more similar to humans than dogs (38). SARS-CoV-2 RNA recovered from hamsters 1-d postinoculation had relatively low-frequency variants compared to dogs and cats (Table 2) but was under strong selective pressure (Fig. 4). The virus recovered from one ferret did not demonstrate the reversion of Vero E6 cell–passaged variants (Fig. 2). However, spike residue 501 was under selection, as has been reported in other mustelids (39–41).

### Variants of Concern.

We detected variation at positions that are implicated in the adaptive evolution of SARS-CoV-2 in humans. Some variants, like spike D614G, are identical to variants from human infections. Others, like spike D215N and N501T, represent different substitutions at the same sites as “variants of concern,” which are termed as such because of their potential effects on viral fitness, transmission, replication, or immune evasion. The rapid emergence of these variants in RNA collected 1- to 3-d postinoculation illustrates substantial adaptive pressures shaping viral evolution in early stages of host switching.

Spike N-terminal domain (NTD) variants H69R, D215N, and D138Y were detected in cats, dogs, and hamsters (Fig. 3*D*). D215N reached highest frequencies in dogs, and H69R went to fixation in Cats 5 and 6. In contrast, D215H was present at fixation in the inoculum (Fig. 2). In humans, the spike mutation D215G is characteristic of the beta variant lineage (B.1.351, South Africa) (42). Position 215 is also adjacent to an insertion in virus recovered from deer mice [*Peromyscus maniculatus* (43)]. D138Y is a defining mutation of the gamma variant lineage (P.1, Brazil) (44).

The frequency of NTD variants reported in humans, and in animals in this study, suggests that this domain is a hotspot for variants with transmission or replication advantages to emerge. It is somewhat surprising that the number of variants identified here exceeded those detected in the receptor-binding domain (RBD; Fig. 3*D*), as RBD-ACE2 interactions resulting in viral entry have been projected to be key determinants for species susceptibility (45, 46). At least one neutralizing human antibody that binds to the NTD has been identified (47), and this domain has been intensely scrutinized as a vaccine epitope. NTD variants are, thus, of concern, as adaptive changes in this region may facilitate immune evasion.

Amino acid 501, asparagine in initial isolates of SARS-CoV-2, is located in the RBD of the spike protein (Fig. 3*D*). This position is a threonine (T) in SARS-CoV and in the closely related coronavirus isolated from a pangolin (48). We observed the rapid emergence of N501T in one ferret and uncovered its occurrence at similarly high frequencies in samples from two additional ferrets through targeted PCR and direct sequencing. The identical variant arose in 11 out of 11 ferrets following experimental infection (39) and has also emerged repeatedly on mink farms in association with 69–70del (40). Thus, these amino acid changes may contribute to the adaptation of SARS-CoV-2 to ferret and mink hosts (41, 49). The cooccurrence of spikes 69–70del and N501Y has been recorded in the alpha variant lineage (B.1.1.7, UK) in humans (25, 50). The N501Y substitution has been shown to increase binding affinity to the human ACE2 (51).

H655Y, a feature of the gamma variant in humans, was detected in RNA from three of six cats, all three dogs, and two hamsters and was present at low frequency (1.1%) in the P3 viral inoculum (Table 1). Spike amino acid 655 is located near the S1/S2 cleavage site of the spike protein between the RBD and the fusion peptide. This variant has been reported to be under positive selection in experimentally inoculated cats (52) and has been shown to emerge following one passage of pseudotyped SARS-CoV-2 in cell culture in the presence of anti-spike human antibodies (53).

Spike variant D614G was the first widely recognized emergent mutation in human SARS-CoV-2 lineages in early 2020 and has rapidly increased in prevalence to become the dominant sequence worldwide. By the end of February 2021, D614G represented 94.8% SARS-CoV-2 sequences publicly available in the GISAID database (54). The growth advantage of this variant is ascribed to replication fitness advantage (26, 55). We identified D614G in two inoculated cats. This variant was not detectable in the inoculum, and thus likely arose and was under selection in cats. The variant reached fixation in Cat 5 and was transmitted to Cat 6 via contact infection, indicating this variant could become established in a cat-to-cat transmission chain. While it is important to consider each amino acid change in SARS-CoV-2 in conjunction with all changes across the viral genome, the detection of specific substitutions in experimentally inoculated animals identical to changes in viral lineages infecting humans may suggest the convergent evolution or the potential for variants to originate in animals followed by spillover back into humans.

Variant L37F in nsp6, detected in RNA from cats, dogs, and hamsters, also emerged early during the COVID-19 pandemic, as a defining mutation of the GISAID Clade V; however, it was not as ultimately successful in spreading in humans as D614G (54). We detected L37F, resulting from a single-nucleotide change in all four species. Two frameshift mutations (nucleotide insertion and nucleotide deletion) were also detected at L37. Although there is no longer sustained public attention on L37F in human SARS CoV-2, previous research has linked the presence of the L37F G > T SNV to asymptomatic infection in humans (56).

Given the susceptibility of companion animals to SARS-CoV-2 infection and reports of SARS-CoV-2 transmission from humans to animals and then back to humans (*SI Appendix*, Table S1), the rapid adaptation we document illustrates the potential of reverse zoonosis to accelerate variant emergence for SARS-CoV-2 and other viruses. The rapidity of in vitro and in vivo adaptation underscores the extraordinary plasticity and adaptive potential of SARS-CoV-2. Pathogens are under strong selective pressure to propagate in the host environment, while host defenses are aligned to prevent pathogen replication. This host–pathogen arms race results in varied outcomes that can lead to increases or decreases in virulence and transmission (57). Thus, monitoring SARS-CoV-2 evolution in hosts with the potential to infect humans should be a high priority, as de novo novel variants or recombinants between human and animal strains could result in altered transmission pathways and vaccine efficacy (58).

Our work additionally illustrates that virus evolution and adaptation following passage in cell culture and experimental infection is far more complex than has typically been acknowledged. The advent of new technologies that afford the opportunity to assess viral quasispecies within inocula and biological samples provides an exciting opportunity for future studies of viral evolution and pathogenesis in both humans and animal hosts.

## Materials and Methods

### Cell Culture Passage In Vitro.

SARS-CoV-2 strain USA-WA1/2020 (GenBank MN985325.1) was obtained (27) and passaged in Vero E6 cells a total of three times. A total of 100 μL viral stock was inoculated onto a flask of confluent Vero E6 cells, allowed to adsorb for 30 min, incubated in cell culture media (Dulbecco’s Modified Eagle Media supplemented with 10% fetal bovine serum and antibiotics) for 3 to 4 d, harvested, and frozen in aliquots.

### Infections In Vivo.

Intranasal inoculations of cats, dogs, and ferrets with SARS-CoV-2 infection were conducted in Animal Biosafety Level 3 facilities at Colorado State University. We conducted a comprehensive analysis of infection outcomes, including the assessment of virus neutralization, seroconversion, cat-to-cat transmission, and resistance to reinfection (6). SARS-CoV-2 strain USA-WA1/2020 was expanded in Vero E6 cells, and all four species were inoculated with the same P3 viral stock. Cats 2, 3, and 4 were in Cohort 1, and Cats 1 and 5 were in Cohort 2. Cat 6 was cohoused with Cat 5 for the contact transmission of the virus (6). Animals were lightly anesthetized and between 10^5^ to 10^6^ pfu SARS-CoV-2 was instilled via the nares. Oropharyngeal and/or nasal samples collected for up to 10 d. Oral swabs were placed in BA-1 medium (Tris-buffered minimum essential media containing 1% bovine serum albumin) supplemented with gentamicin, amphotericin B, and penicillin/streptomycin. Nasal flushes were performed by instilling 1 mL BA-1 dropwise into the nares of awake or lightly anesthetized cats, dogs, and ferrets and collecting nasal discharge into a sterile Petri dish by allowing the wash fluid to be sneezed out or dripped onto the dish. Viral titers (expressed as plaque-forming units) were assessed for all infected individuals (6).

### RNA Extraction and Sequencing.

A total of 100 μL lavage fluid was added into 900 μL trizol to inactivate the virus and prepare the sample for RNA extraction. RNA was extracted using a modified Zymo RNA Clean and Concentrator 5 kit (Zymo Research). Samples were thawed on ice, and 200 μL chloroform was added. Samples were agitated by hand for 15 s, incubated at room temperature for at least 2 min, and centrifuged for 10 min at 12,000 *g*. A total of 450 μL of the aqueous phase was removed and placed in 1.5-mL microcentrifuge tubes containing 900 μL 1:1 Zymo RNA-binding buffer and 100% ETOH (450 μL each). Samples were vortexed, and then, 750 μL of each was transferred to RNA CC-5 columns and centrifuged for 1 min at 12,000 *g*. Each column was washed with 400 μL Zymo RNA wash buffer and centrifuged again for 1 min at 12,000 *g*. A mixture of 24 μL Zymo RNA wash buffer, 3 μL DNase I buffer, and 3 μL DNase I (both New England Biolabs) was added to 30 μL of the mixture and incubated for 22 min at room temperature. Following incubation, columns were spun in collection tubes for 30 s at 9,000 *g*. RNA prep buffer (400 μL) was added to each column and centrifuged again. Flow through was discarded and columns washed with 800 μL RNA wash buffer, followed immediately by a second wash with 400 μL. Wash buffer was discarded, and samples were centrifuged for 1 min at 16,000 *g* to dry the membrane. We transferred columns to labeled, sterile microcentrifuge tubes and to elute RNA. Immediately following RNA extraction complementary DNA was generated by adding 1 μL of 3μg/μL of random primers (Thermo Fisher Scientific) and 1 μL of 10 mM dNTPs (New England Biolabs) to 10 μL RNA and incubating at 65 °C for 5 min on a C1000 Touch Thermal Cycler (Bio-Rad), followed by chilling on ice. We added 4 μL 5× First-Strand Buffer, 2 μL of 0.1 M DTT, and 1 μL RNase OUT (40 units/μL) to each sample, pipette mixed, and incubated at 25 °C for 2 min. A total of 1 μL (200 units) SuperScript II RT was added, pipette mixed, and incubated at 25 °C for 10 min, 42 °C for 50 min, and 70 °C for 15 min. cDNA was frozen at −20 °C until use in library preparation.

### Amplicon Generation and Library Preparation.

We employed an amplicon-based next-generation sequencing approach using the primers and protocols developed and optimized for SARS-CoV-2 by the ARTIC Network (60, 61). All samples were sequenced with ARTIC version 3 primers except for Cat 5 and one replicate of Cat 4, which were sequenced with the previous version 2 primers. This sequencing method generates high coverage of viral genomes from even low-template samples by utilizing an initial PCR step resulting in ∼400 bp amplicons and primers that span the coding region (60). Briefly, two primer pools were used for the initial PCR amplification and then pooled prior to library preparation. We then visualized PCR products on agarose gels and quantified them with the Qubit Broad Range kit (Thermo Fisher Scientific). Samples from Ferret 2 and Ferret 3 were not sufficiently amplified for downstream next-generation sequencing. We normalized samples to 540 ng cDNA. We prepared sequencing libraries using the New England Biolabs Ultra II kit, with the only modification being the use of Ampure XP beads for cleanup and size selection (Beckman Coulter). Following adapter ligation, a single size selection was conducted at 0.65×. We then pooled libraries for sequencing on Illumina MiSeq (Illumina) using the version 2 500-cycle 2 by 250-bp kit.

### Targeted PCR and Cloning for Spike Variant N501T.

We designed a targeted PCR for the region of the spike gene containing N501T to amplify cDNA from the two additional ferrets in our study that did not adequately amplify with ARTIC primers. PCR amplification was carried out according to the manufacturer’s protocol for PlatinumTM SuperFiTM PCR Master Mix (Thermo Fisher Scientific) using the following primer set: forward, AGGCTGCGTTATAGCTTGGA and reverse, CTGTGGATCACGGACAGCAT. Products were visualized on agarose gels and purified using the MEGAquick-spinTM Plus Total Fragment DNA Purification Kit (iNtRON Biotechnology), prior to being ligated into pJET1.2/blunt vectors and cloned using a CloneJet PCR cloning kit (Thermo Fisher Scientific). Plasmids were purified from Ferret 2 (*n* = 6 clones) and Ferret 3 (*n* = 3 clones) with the DNA-Spin Plasmid Purification Kit (iNtRON Biotechnology), according to the manufacturer’s protocol, and direct sequenced in both directions (Psomagen, Inc.). Sequences were aligned to the USA-WA1/2020 reference sequence and interrogated at position 23,064 for the presence of the A > C single-nucleotide change that causes spike N501T.

### Bioinformatics and Data Analysis.

Raw sequencing data were input into a comprehensive Nextflow pipeline for processing next-generation sequencing data and SNV and SV calling. Nextflow is a bioinformatics workflow manager that facilitates the development of complex and reproducible computational pipelines (62). Briefly, data were trimmed for adapters and low quality using Cutadapt (63), followed by aligning reads to the viral reference sequence. Data were preprocessed for quality with GATK (64), prior to calling SNVs and SVs with LoFrEq (65). SnpEff and SnpSift were used to annotate variants and predict their functional effects (66, 67). We designed our variant-calling pipeline to ensure that we could differentiate between variants that were not detected because of their absence or presence at a frequency below our detection threshold, as compared to the inadequacy of coverage depth. The outputs of these analyses were tabulated, processed, and visualized in R.

University of California, San Francisco (UCSF) Chimera software was used to visualize the location of variants in the three-dimensional structure of the SARS-CoV-2 spike protein (68). SNPGenie was used to calculate nonsynonymous and synonymous nucleotide diversity (69). SNPGenie takes a population of sequences and estimates the mean number of pairwise differences per site or the nucleotide diversity. Estimates are weighted by allele frequencies. The SNPGenie snpgenie.pl takes as input variant call information contained in .vcf files, a reference genome and genome annotations, and outputs estimates of nucleotide diversity at nonsynonymous and synonymous coding sites at both a genome/population level and by gene product. One advantage of using the nucleotide diversity statistic (π) to compare intrahost viral diversity is that it is not biased by variations in sequencing depth (70).

## Data Availability

All SARS-CoV-2 raw sequence data used in this study are publicly available in the National Center for Biotechnology Information (NCBI) Sequence Read Archive (SRA) database under BioProject PRJNA704947 (71). The bioinformatics pipeline used to analyze the data is publicly available at https://github.com/stenglein-lab/viral_variant_caller. The code used to create the three-dimensional visualization of the spike protein is available at https://github.com/stenglein-lab/highlight_residues_on_spike_structure. Additional data, including variant summary tables output by the pipeline and R scripts for data processing and visualization, are available at https://github.com/laurabashor/SARS-CoV-2-evolution. All other study data are included in the article and/or *SI Appendix*.

## Change History

November 9, 2021: The license for this article has been updated.
